# Areas of consensus on unwarranted and warranted transfers between nursing homes and emergency care facilities in Norway: a Delphi study

**DOI:** 10.1186/s12913-024-10879-3

**Published:** 2024-03-26

**Authors:** Arne Bastian Wiik, Malcolm Bray Doupe, Marit Stordal Bakken, Bård Reiakvam Kittang, Frode Fadnes Jacobsen, Oddvar Førland

**Affiliations:** 1 Centre for Care Research, West. Western, Norway University of Applied Sciences, Bergen, Norway; 2 Max Rady College of Medicine, University of Manitoba, Winnipeg, Canada; 3 National Centre for Emergency Primary Health Care, NORCE Norwegian Research Centre, Bergen, Norway; 4 University of Bergen, Bergen, Norway; 5 Department of Medicine, Haraldsplass Deaconess Hospital, Bergen, Norway

**Keywords:** Delphi study, Emergency care, Nursing home, Patient Transfer

## Abstract

**Background:**

Transferring residents from nursing homes (NHs) to emergency care facilities (ECFs) is often questioned as many are terminally ill and have access to onsite care. While some NH to ECF transfers have merit, avoiding other transfers may benefit residents and reduce healthcare system costs and provider burden. Despite many years of research in this area, differentiating warranted (i.e., appropriate) from unwarranted NH to ECF transfers remains challenging. In this article, we report consensus on warranted and unwarranted NH to ECF transfers scenarios.

**Methods:**

A Delphi study was used to identify consensus regarding warranted and unwarranted NH to ECF transfers. Delphi participants included nurses (RNs) and medical doctors (MDs) from NHs, out-of-hours primary care clinics (OOHs), and hospital-based emergency departments. A list of 12 scenarios and 11 medical conditions was generated from the existing literature on causes and medical conditions leading to transfers, and pilot tested and refined prior to conducting the study. Three Delphi rounds were conducted, and data were analyzed using descriptive and comparative statistics.

**Results:**

Seventy-nine experts consented to participate, of whom 56 (71%) completed all three Delphi rounds. Participants reached high or very high consensus on when to *not* transfer residents, except for scenarios regarding delirium, where only moderate consensus was attained. Conversely, except when pain relieving surgery was required, participants reached low agreement on scenarios depicting warranted NH to ECF transfers. Consensus opinions differ significantly between health professionals, participant gender, and rurality, for seven of the 23 transfer scenarios and medical conditions.

**Conclusions:**

Transfers from nursing homes to emergency care facilities can be defined as warranted, discretionary, and unwarranted. These categories are based on the areas of consensus found in this Delphi study and are intended to operationalize the terms warranted and unwarranted transfers between nursing homes and emergency care facilities.

**Supplementary Information:**

The online version contains supplementary material available at 10.1186/s12913-024-10879-3.

## Background

The prevalence of dementia and frailty is high and continues to increase amongst Norwegian nursing home (NH) residents [1], which increases their risk of experiencing adverse health events during transfers to emergency care facilities (ECFs) for acute medical reasons [2, 3]. Two Norwegian studies have shown that the incidence of these transfers is about 600 per 1000 NH beds annually [4, 5], and according to an international systematic review, there is no agreed upon strategy to determine which of these NH to ECF transfers are appropriate [6]. Such transfers are not only dependent on a NH resident’s health but are also influenced by conditions within the healthcare system. In this study we present the results of a Delphi study designed to examine consensus across Norwegian medical professionals regarding the appropriateness of different NH to ECF transfer scenarios.

### Comparisons and context regarding the Norwegian setting

In Norway, general practitioners (GPs) provide an essential first line of medical care and together with out-of-hours (OOH) emergency health care centers, these professions provide strict ECF gatekeeping for all Norwegians including NH residents. NH residents, including those facing potentially life-threatening situations, should be assessed by a GP before admission to ECF [7, 8]. All municipalities in Norway must ensure that inhabitants have appropriate access to OOH emergency primary health care [9]. Types of ECF offered in Norway include OOH centers, hospital emergency departments (EDs) and municipal in-patient acute care units (MAU) [10], and municipalities are required to provide comparable standards of care and medical follow-up throughout the country [11].

In 2019, dementia and falls were the two most expensive health conditions for the Norwegian health care system, representing 10.2% and 4.6% of estimated total health care spending, respectively [12]. According to OECD data, Norway ranks third highest in long-term care (LTC) spending per capita [13]. While the number of Norwegians 80 + years old living in LTC institutions has declined in recent years (from 26,913 in 2009 to 21,662 in 2022) [14], the size of this population has increased during this same period (219 500 people in 2009, 246 000 people in 2022) [15]. NH (with short-term and long-term beds), home health care, GPs and OOH services are provided and administered at a municipal level in Norway. Hence, most decisions about NH to ECF transfers occur at the municipal level.

## Methods

### Overview and scenario statement process

Delphi methods are used to synthesize current knowledge and agreement from a diverse group of experts, using iterative rounds of questionnaire completion followed by aggregate group feedback permitting Delphi participants to amend their initial responses [16]. An e-Delphi was used to offer independent and anonymous participation with no requirement for face-to-face meetings [17]. Our Delphi was created to generate consensus across expert providers diversified by training and healthcare environment (e.g., NHs, ECFs), on scenarios of warranted and unwarranted NH to ECF transfers.

The selection of 12 scenarios and 11 medical conditions to be questioned came from a process where we started with a wide range of causes and medical conditions discussed as influencing potentially warranted and unwarranted transfers [5, 18–21] and defined two general statements regarding life expectancy and life quality. We then filtered the scenarios and medical conditions through a pilot-panel with two RNs and two MDs experienced in evaluating NH to ECF transfers. These experts recommended and removed questions based on their experiences. They were not included in the study panel. The author-group then selected from this revised list the scenarios and medical conditions to be in the Delphi questionnaire. The final list of scenario questions used in this research is presented in Table 1. The final list of medical conditions is presented in Table 2.

**Table 1:** Scenario questions on transfers between Nursing Homes and Emergency Care Facilities in Norway

**Take as a starting point the situation in the norwegian health care system in 2023 as you know it. how much do you agree with the following statements: transfer of a patient with a long term care stay in a nursing home to an emergency care facility should mainly not happen:**
When a transfer reduces residents expected lifetime significantly
When a transfer reduces life quality for the resident in the long run
When a non-transfer preference was expressed in preliminary talk at the NH
When a transfer has significant delirium risk attached to it
When the resident expresses transfer is not wanted
When next of kin expresses transfer is not wanted
When the resident is in a palliative state
**Take as a starting point the situation in the norwegian health care system in 2023 as you know it. how much do you agree with the following statements: transfer of a patient with a long term care stay in a nursing home to an emergency care facility should mainly happen:**
When the condition remain unclear after MD assessment at the Nursing Home
When the residents condition was good before the incidence with acute functional decline occured
When next of kin expresses that a transfer is wanted
When the resident expresses that a transfer is wanted
When a transfer for a surgical operation could be pain-reliving for the resident

**Table 2:** Medical conditions in survey on transfers between nursing homes and emergency care facilities in Norway

Consider the following medical conditions: amongst long term care residents in nursing homes, where should they predominatly be treated and/or assesed at?	Answer options:
Hip fracture	Binary option: NH or ECF
Heart attack	Binary option: NH or ECF
Anemia	Binary option: NH or ECF
Pneumonia	Binary option: NH or ECF
Stroke	Binary option: NH or ECF
Urinary retention	Binary option: NH or ECF
Sepsis	Binary option: NH or ECF
Urinary tract infection	Binary option: NH or ECF
Unintentional weight loss	Binary option: NH or ECF
Other fractures	Binary option: NH or ECF
Dehydration	Binary option: NH or ECF

### Selection criteria for experts to be involved and recruitment

The panel of expert participants for the Delphi was selected from those considered to have professional expertise relevant to the research question [22]. Delphi participants must have been working as an RN or MD with NH residents in an ECF or NH between 2017 and 2023. Participants were asked to identify if they were within the eligibility criteria once they received the invitation.

The Delphi expert panel was recruited during April 2023. Participants were recommended from key Norwegian organizations such as Centers for Development of Institutional and Home Care Services, labor unions for RNs and MDs, academic researchers within the field of gerontology and geriatrics, and research advisors in the larger municipalities. In total 344 experts were suggested, of whom 125 were contacted based on a desire for variations regarding geography, gender, and profession. RNs and MDs, who are also occupied with research and teaching, were included as experts.

### Survey development and application

The Delphi method used in this research is based on the CREDES guidance on Conducting and Reporting Delphi Studies [23]. Participation fatigue and the reduction of feedback is considered to increase after three rounds [24], so the participants were informed that only three rounds would be undertaken. Non-respondents in one round were not allowed to participate in subsequent rounds.

**Fig. 1:**
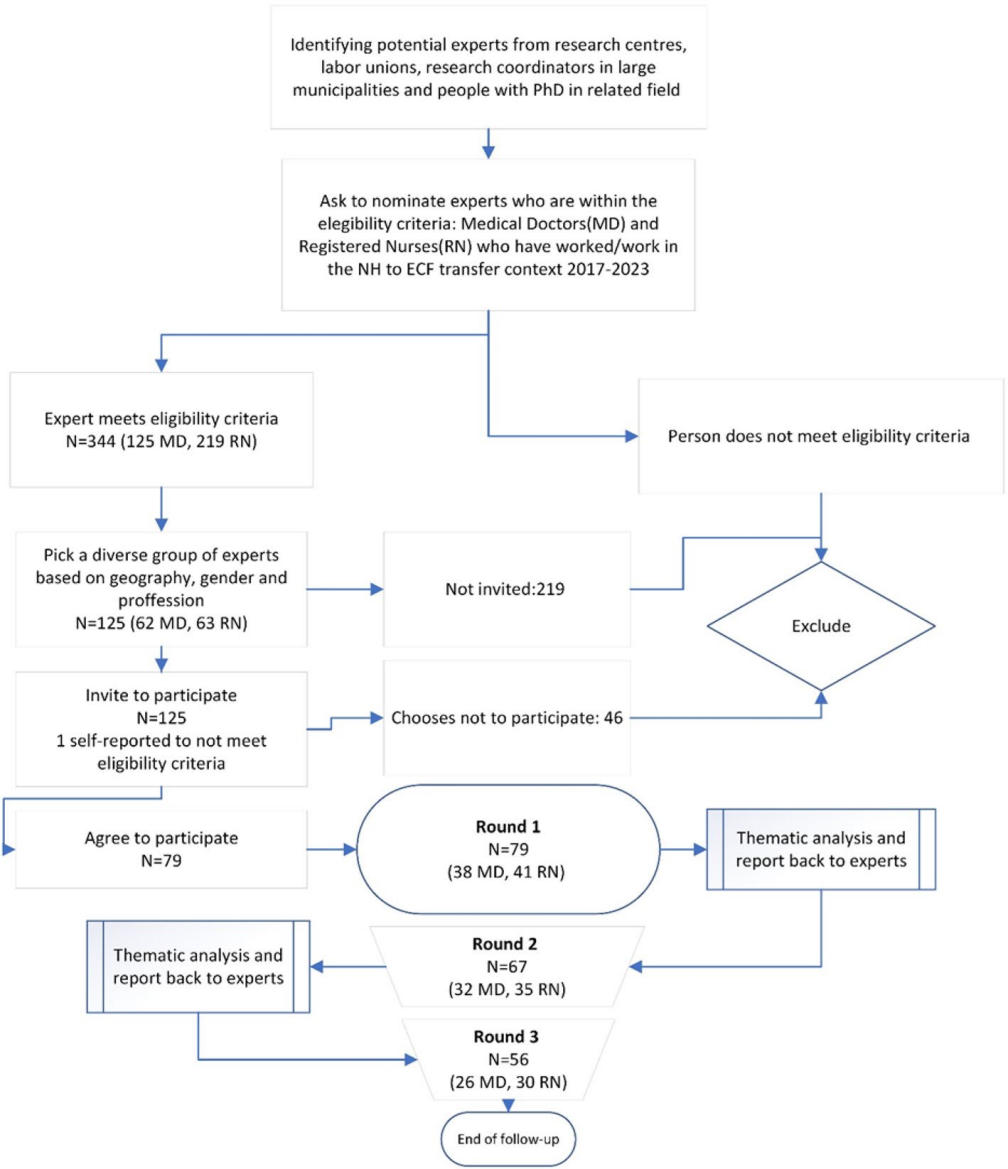
Flowchart of Delphi process to assess nursing home to emergency care facility transfers

The final questionnaire consisted of four sections: self-provided information about the participant, seven statements on when not to transfer from NH, five statements on when to initiate transfer from the NH, and eleven specific medical conditions where the participants were given a binary preference option of either transferring from NH or not (Fig. 1).

### Delphi scoring and data analysis

Participants rated their agreement on a Likert scale from 1 (not agreeing with this statement) to 9 (completely agreeing with this statement). A score of 7 and above defined agreement and results from each Delphi round were analyzed according to the method prescribed by Van der Steen et al. [25]. Measures of central tendency (median) and variation (interquartile range, IQR) were employed to set the levels of consensus:

- *Very High* (80% + of participants provided a score ≥ 7; IQR = 0).
- *High* (80% + of participants provided a score ≥ 7; IQR ≤ 2).
- *Moderate* (60% + of participants provided a score ≥ 7; IQR ≤ 4).
- *Low* (All other results).

Consensus on a statement was defined as participants reaching a “high” or “very high” consensus [26]. As recommended by others the consensus was defined as stable if less than 15% of participant scores changed tertials (1–3, 4–6, 7–9) between rounds [27]. Logistic regression analysis was used to find if one group had significantly different answers on the statements and we use a significance level of 0.05. For analyzing quantitative data, STATA 17.0 was used.

**Fig. 2:**
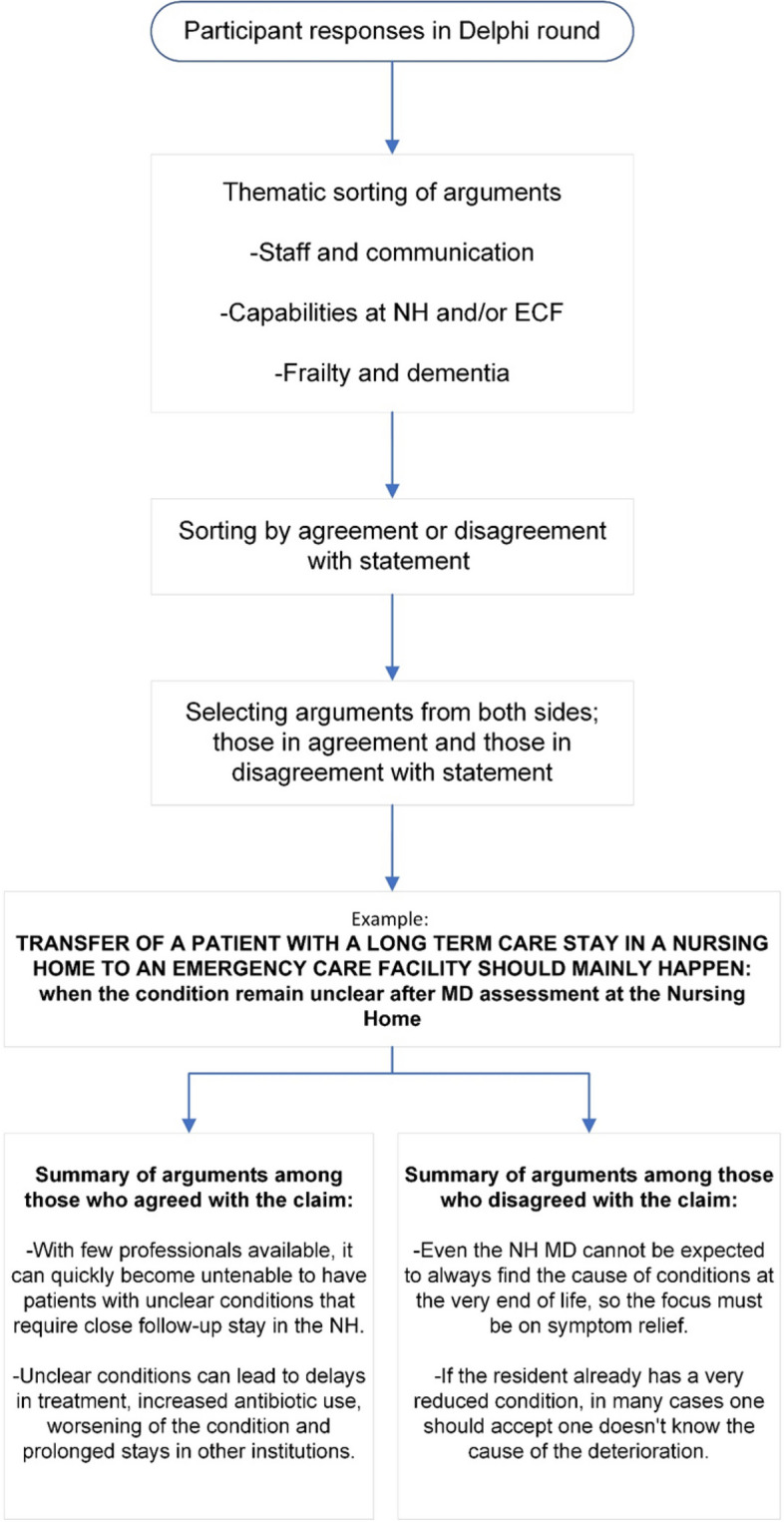
Flowchart of how arguments in Delphi on NH-ECF transfers were summarized back to participants

The comments on the statements were thematically analyzed [28] and summarized for the second and third round. Original wording, when not containing identifying elements, from one of the participants that represented the wording of others with similar arguments was used whenever possible [29]. An example of this process is shown in the flow chart in Fig. 2.

### Research ethics

The research was assessed and recommended by the Norwegian Agency for Shared Services in Education and Research (SIKT), reference number 986883. The legal basis was informed consent under General Data Protection Regulation art. 6 nr.1a. Informed consent was obtained for all participants through e-Delphi on the SurveyXact platform. This study is part of the larger Knowledge to Action (K2A) project funded by the Research Council of Norway, with the aim to investigate transfers between NH and ECF.

## Results

### Study participation

On April 26th, 2023, the 125 experts were given the option to participate via a link to the first round, to decline, or to state that they did not fit the inclusion criteria. One person self-reported as not fitting the inclusion criteria. Out of the 124 remaining participants, 79 (64%) completed the first Delphi round, whereas 67 participants and 56 participants completed rounds 2 (launched May 8) and 3 (launched May 30) of the Delphi survey, respectively. A total of 56 participants completed all three Delphi rounds, resulting in an average response rate of 77.8% between rounds 2 and 3 as compared to round 1 participants.

As shown in Table 3, 41 RNs and 38 MDs participated in the first round of this study. RNs were predominantly female, and MDs were split somewhat equally amongst the genders in the rounds. More than half of the nurses had a NH background, while MDs had a mixed or emergency care background. In the first round, 42% of the MDs reported working in both local municipal NHs and in various forms of acute care and community hospitals. Of the RNs, 29% in the first round reported working at MAUs, OOH clinics or other forms of intermediary local acute care services.

**Table 3:** Participant characteristics in Delphi on unwarranted and warranted nursing homes and emergency care facilities transfers

	First round	Second round	Third round
Expert panel participants	79	67	56
**Registered Nurse (RN) n (%)**	**41 (52%)**	**35 (52%)**	**30 (54%)**
Participant sex (Male) (% of RN)	3 (7%)	3 (9%)	3 (10%)
Centrality Index			
Level 1 (Most urban)	4 (10%)	3 (9%)	3 (10%)
Level 2	7 (17%)	6 (17%)	6 (20%)
Level 3	14 (34%)	13 (37%)	10 (33%)
Level 4	6 (15%)	4 (11%)	4 (13%)
Level 5	6 (15%)	5 (14%)	5 (17%)
Level 6 (Most rural)	4 (10%)	4 (11%)	2 (7%)
Nursing Home background	26 (63%)	22 (63%)	19 (63%)
Mixed background	12 (29%)	10 (29%)	8 (27%)
Acute Care background	3 (7%)	3 (9%)	3 (10%)
**Medical Doctor (MD) n (%)**	**38 (48%)**	**32 (48%)**	**26 (46%)**
Participant sex (Male) (% of MD)	16 (42%)	14 (44%)	12 (46%)
Centrality Index			
Level 1 (Most urban)	9 (24%)	9 (28%)	7 (27%)
Level 2	16 (42%)	12 (38%)	9 (35%)
Level 3	8 (21%)	6 (19%)	6 (23%)
Level 4	2 (5%)	2 (6%)	2 (8%)
Level 5	2 (5%)	2 (6%)	1 (4%)
Level 6 (Most rural)	1 (3%)	1 (3%)	1 (4%)
Nursing Home background	8 (21%)	8 (25%)	8 (31%)
Mixed background	16 (42%)	12 (38%)	10 (38%)
Acute Care background	14 (37%)	12 (38%)	8 (31%)

Statistics Norway uses a centrality index to score the rurality of Norwegian municipalities based on inhabitants’ road-distance to public services and workplaces [30]. The RNs participating in the Delphi were more rural and the MDs less rural than the general population. The participants came from 36 municipalities in 14 of the 15 counties in Norway.

### Transfer preferences for medical conditions

Table 6 shows a list of acute care transfer choices for specific medical conditions, based on a list from 2005 [19], presented with a strict binary option on whether or not to transfer to ECF. In this, there was complete agreement on predominantly transferring long-term care NH residents for hip fracture. In the same list, there was complete agreement on predominantly not transferring long-term care NH residents for dehydration (100%), urinary tract infection (100%), pneumonia (99%), urinary retention (96%) and severe weight loss (94%). Disagreement was found in preferences for LTC resident transfer when it came to sepsis (36%), stroke (29%), severe anemia (46%), and heart attack (35%). A significantly lower preference was found to send patients with a myocardial infarction to emergency care facilities in more urban municipalities and amongst the MDs. The authorship team decided to exclude medical conditions from subsequent Delphi rounds given; (1) the limited number of dissenting arguments from participants for these conditions, and (2) the high volume of written comments for the additional scenarios. Survey items were reduced in subsequent Delphi rounds to avoid participant burnout and to help optimize response rates.

### Consensus on statements for and against the transfer of residents

Tables 4 and 5 show consensus was reached on 7 out of 12 statements in the third round. Consensus was mostly high or very high about scenarios depicting when to *not* transfer NH residents. The exception was a low consensus when it came to not transferring based on delirium risk and regarding the next-of-kin wanting their relative to stay put, as shown in Table 4.

**Table 4:** Delphi survey on transfers between nursing homes and emergency care facilities in Norway

Delphi factor	First round			Second Round				Third Round				
	% of score ≥ 7	IQR	Consensus Level	% of score ≥ 7	IQR	Consensus Level	Stability	% of score ≥ 7	IQR	Consensus Level	Stability	*P*-value < 0.05
*Transfer ****should not**** happen when:*												
It reduces residents expected lifetime significantly	**94%**	**0**	**Very high**	**97%**	**0**	**Very high**	**Yes**	**100%**	**0**	**Very High**	**Yes**	
It reduces life quality in the long run	**87%**	**1**	**High**	**95%**	**0**	**Very high**	**Yes**	**95%**	**1**	**High**	**Yes**	
Non-transfer preference in preliminary talk	**98%**	**1**	**High**	**97%**	**1**	**High**	**Yes**	**98%**	**1**	**High**	**Yes**	
Significant delirium risk attached	75%	3	Moderate	86%	2	**High**	**No**	**75%**	2	Moderate	No	Gen (1,3) (-)
												MD (3)(-)
Resident expresses transfer is not wanted	**87%**	**1**	**High**	**94%**	**1**	**High**	**Yes**	**96%**	**1**	**High**	**Yes**	
Next-of-kin expresses transfer not wanted	73%	3	Low	77%	1	Low	No	63%	1	Low	No	
Resident is in a palliative state	96%	0	**Very high**	**97%**	**0**	**Very high**	**Yes**	**98%**	**0**	**Very High**	**Yes**	

Table columns: round, consensus (% of score ≥ 7), inter-quartile range *IQR*, stability between the round and the previous one, and significant differences (*P*-value < 0.05) in answers by panel characteristics gender *Gen*, profession *MD* or rurality *Urb*

Bold font: consensus

Minus (-) indicates lower agreement, ( +) expresses higher agreement with the statement

Gender (Gen) has male set as 1

Urbanity (Urb) is a Statistics Norway measure of rurality

The questions are ranked in the order they were presented in the project proposal pre-randomized Delphi-survey

Logistic regression was used to determine significant differences in coefficients

**Table 5:** Delphi survey on transfers between nursing homes and emergency care facilities in Norway

Delphi factor	First round			Second Round				Third Round				
	% of score ≥7	IQR	Consensus Level	% of score ≥7	IQR	Consensus Level	Stability	% of score ≥7	IQR	Consensus Level	Stability	*P*-value <0.05
*Transfer ****SHOULD**** happen when:*												
Condition unclear after MD assessment	43 %	4	Low	40 %	2	Low	No	45 %	3	Low	Yes	MD (1,2,3) (-)
Condition was good before acute function decline	57 %	4	Low	81 %	1	**High**	**No**	**83 %**	**1**	**High**	**Yes**	MD (1)(-)
Next-of-kin expresses transfer wanted	22 %	3	Low	4 %	1	Low	No	12 %	3	Low	No	
Resident expresses transfer wanted	47 %	3	Low	45 %	2	Low	No	34 %	2	Low	Yes	Gen (2,3) (-) Urb (1)(-)
A surgical operation could be pain-reliving	**90 %**	**1**	**High**	**97 %**	**0**	**Very high**	**Yes**	**100 %**	**0**	**Very high**	**Yes**	

Table columns: round, consensus (% of score ≥7), inter-quartile range (IQR), stability between the round and the previous one, and significant differences (*P*-value <0.05) in answers by panel characteristics gender (Gen), profession *MD* or rurality *Urb*

Bold font: consensus

Minus (-) indicates lower agreement, (+) expresses higher agreement with the statement.

Gender *Gen* has male set as 1

Urbanity *Urb* is a Statistics Norway measure of rurality.

The questions are ranked in the order they were presented in the project proposal pre-randomized Delphi-survey

Logistic regression was used to determine significant differences in coefficients

Alternatively, participants generally reached low agreement on scenarios depicting when NH to ECF transfers should occur. As exceptions, participants unanimously agreed that NH to ECF transfers should occur for pain-relieving surgery (e.g., for hip fractures). There was significant disagreement between the RNs and MDs on whether to transfer after an inconclusive assessment by an MD. The participants with an MD background were more critical to transfers under these circumstances, while RNs were more likely to suggest a transfer. 

### Delphi findings

The results of the Delphi are shown in Tables 4, 5 and  6.

**Table 6:** Norwegian registered nurses and medical doctors on when to predominantly transfer nursing home long-term-care residents

Condition	Predominantly at the Nursing Home	RN(*n*=41)	MD(*n*=38)	Predominately at Emergency Care Facility	RN(*n*=41)	MD (*n*=38)	Coeff.
Hip fracture	4%(3)	3	0	**96%(76)**	38	38	
Heart attack	35%(28)	5	23	65%(51)	36	15	Urb***(-) MD***(-)
Anemia	46%(36)	17	19	54%(43)	24	19	Gen**(-)
Pneumonia	**99%(77)**	40	37	1%(1)	0	1	
Stroke	29%(23)	7	16	71%(55)	34	21	Gen***(-)
							MD***(-)
Urinary retention	**96%(76)**	38	38	4%(3)	3	0	
Sepsis	36%(28)	9	19	64%(50)	32	18	
Urinary tract infection	**100%(79)**	41	38	0%(0)	0	0	
Unintentional weight loss	**94%(74)**	37	37	6%(5)	4	1	
Other fractures	74%(58)	31	27	26%(20)	10	10	
Dehydration	**100%(79)**	41	38	0%(0)	0	0	

Bold font: consensus

Minus (-) indicates treated/assessed more predominantly at Nursing Home, (+) expresses a preference for transferring to Emergency Care Facilities.

Gender has male set as 1.

*Urb* statistical measure of rurality where 1000 is most urban, 0 least.

*MD* medical doctors, *RN* Registered nurses.

Logistic regression was used to determine significant differences in coefficients (*P*-value <0.05) by gender and centrality.

### Medical condition transfer preference (Table 6)

#### Differences among participants and non-participants in the third Delphi round

We compared the experts who participated (*n* = 56) in the last round and those who had dropped out by then (*n* = 23). There was no significant difference in consensus statement scores between these groups. Full study participants were more likely to be male and less likely to be urban MDs, as compared to partial study participants.

## Discussion

In the present study, both the data and the commentary reflected a consensus with high or very high agreement on a non-transfer regime in most instances. Strict gatekeeping by MDs was idealized. However, some comments suggested this regime was not always practiced and the answers might reflect an ideal, rather than reality practiced in care settings.

NH to ECF transfers is a topic with considerable interest internationally [6] due to the medical uncertainties and ethical considerations associated with transferring high risk and often terminally ill residents. As a result, several Delphi studies have been conducted to find consensus on transfers [31–35]. There has been a lack of studies on warranted and unwarranted transfers from NH to ECF in Norway since 2013 [2, 18]. One Norwegian study left the question of unwarrantedness completely to the referring physicians, by indicating to what degree other care alternatives could have prevented the referral to an OOH primary care center [36]. There have been large changes following from the 2012 implementation of the Coordination Reform [37] where municipalities are now fined for not transferring residents back to LTC after hospital discharge [38].

### Agreement among the expert panel

Answers and comments reflected a unanimous exception from the non-transfer regime for pain-relieving surgery such as for hip-fractures, strongly suggesting these as warranted transfers when they occur. This was also reflected when it came to the conditions, where 96% considered that hip fractures should be treated predominantly in hospital. Transfer for pain-relieving surgery was defined as being warranted, even for people living with severe frailty, including LTC residents with short life expectancy. This is in clear contrast to other medical conditions and situations, where one should either obviously avoid transfers or use discretion.

Acute functional decline was the only statement where the participants went from low consensus to high consensus, with stability between the two last rounds. There were significant differences in the first round, with MDs expressing a lower consensus with the statement than RNs. This result is not in line with a strict no-transfer regime. A key aspect of Norwegian NH to ECF transfers is the role gatekeeping physicians have regarding access to unplanned secondary care outside the institution. Even though a previous report on individual GPs' prior admission regime for the general population showed to be strongly associated with unplanned hospital admissions, the effect on the 30-day risk of death was not significant [39].

### Differences in the expert perspectives

In certain statements, participants expressed significantly different transfer preferences. MDs expressed more hesitancy to transfer in unclear diagnostic situations, whereas RNs expressed that it could be difficult to avoid transferring for diagnostic clarification under circumstances without clear plans of action being set forward by daytime/workday physicians.

The commentary given during the Delphi rounds had a high engagement on next-of-kin involvement. The widest inter-quartile ranges were found in statements concerning the role of next-of-kin. Next-of-kin advocacy can be a source of conflict [40], but there is at the same time a wish for more next-of-kin involvement and volunteering. Aging in place has become a widely adopted public policy in Norway, utilizing resources such as informal caregivers closer to the home before residents enter the LTC, thus providing extensive information on unmet care needs for the resident [41]. This focus on next-of-kin involvement pre-LTC might guide decisions regarding the residents after entering NHs. The number of comments and engagement when it came to next-of-kin interactions and conflicts was higher than for the other statements in the Delphi. The concept of Shared Decision Making (SDM) is “a collaborative process in which patients and providers make health care decisions together, taking into account the best evidence available, as well as the patient’s values and preferences” [42], in a NH setting SDM will often involve next-of-kin involvement. There are three factors necessary for when the clinical scenario is appropriate for SDM: Clinical uncertainty, decision-making ability of the resident and/or their next-of-kin, and sufficient time. The SDM framework align with the comments made by Delphi participants. There are some medical conditions that the panel did not have a clear consensus on whether to predominantly transfer to an ECF or not, highlighting areas where SDM may be especially valuable, since it is better used for problems involving medical uncertainty [43]. According to Romøren et. al. [44] NH MD involvement in end-of-life decisions in Norway, often appear arbitrary and influenced by independent factors differing from resident and next-of-kin values and interests. Conflicts between medically based recommendations and next-of-kin might increase the risk for MDs becoming more concerned about the next-of-kin opinions than the best interest of the resident [45]. The gender-difference found in the Delphi is in alignment with a study from Ringberg et al., where Norwegian female MDs referred more often to reassure the patient and/or next-of-kin and due to perceived deficient medical knowledge [46].

We found a significant difference regarding rurality in the “resident expresses transfer is wanted” statement in the first round. There was low consensus on transferring if the resident did not wish so. Several of the experts voiced that long travel distances could be a reason for not transferring after listening to next-of-kin or the resident, noting that most residents preferred to remain in the NH for the final part of their life. Few participants suggested in their comments or consensus statements that the hospitalization-rate should increase with the level of rurality, contrary to the findings in previous studies [47, 48]. Healthcare workforce turnover was an area of importance according to several of the rural experts. There were several participants located in municipalities of the traditional territories contemporarily inhabited by Sámi people.

Delirium during a hospitalization or ECF encounter is associated with poor outcomes and problematic discharges for older adults [49, 50]. The delirium risk was by some of our participants regarded as unavoidable for most NH residents. The statement regarding delirium risk went from moderate consensus in the first round to high consensus in the second, to then fell back to the initial moderate consensus in the final round with no stability between the rounds. Hence, stable agreement was not observed regarding this statement.

To operationalize the terms of warranted and unwarranted transfers in a Norwegian NH setting, we suggest a three-way split of the concept, where statements without consensus in the Delphi might be relegated to a discretionary group. The two initial statements regarding life quality and life expectancy, that by nature were too general to be used in registry data later, were left out. By splitting our data in three groups, we intend to summarize and operationalize our findings for the health and care sector and as a background for further, scientific analysis (Table 7).

**Table 7:** Summary of consensus regarding warranted and unwarranted transfers between nursing homes and emergency care facilities

Warranted	Discretionary	Unwarranted
For pain-reliving surgical operations.	Delirium risk	Non-transfer preference in preliminary talk
For acute functional decline	Next-of-kin advocacy	Resident expresses transfer not wanted
	Condition unclear after MD assessment	In a palliative state
	Anemia	Pneumonia
	Heart attack	Urinary retention
	Stroke	Urinary tract infection
	Sepsis	Unintentional weight loss
	Other fractures	Dehydration

### Further research

Future research should investigate the interplay between conditions, next-of-kin involvement, practice and (un)warranted transfers. It would be of interest to investigate perspectives from residents, next-of-kin and other involved in a future Delphi study.”

### Strengths and limitations

We present data only from the Norwegian health care system. Differences in the organization of the health care system and available resources across jurisdictions might limit the extent that our results can be generalized to other healthcare settings. We did not include residents and next-of-kin in the expert panel since they witness a smaller number of transfers compared to the professionals and are less likely to be familiar with the multitude of scenarios, the language and medical conditions outlined. The proportion of RNs with ECF background participating in the Delphi was lower than hoped for when selecting the panel to be contacted.

The dropout rate from first to second, and second to third rounds was 15% and 16%, respectively, and 29% from first to last round. We also chose to limit the amount of participant background information requested. This was done under the assumption that detailed background information, such as hierarchical information on age, years worked, specialist training, size of unit etc. could lead to social pressure and conformity to a dominant view [23]. Furthermore, while expert opinion is considered to provide a lower hierarchy of ‘best-practice’ evidence [51], defining warranted NH to ECF transfers from the perspective of experts with hands-on knowledge increases the content validity of study results [52]. However, answers might reflect an ideal, rather than reality.

## Conclusions

We found mostly very high consensus, with stability between the rounds for not transferring LTC residents. Statements on when to transfer often had low agreement, with a clear exception for transfers regarding pain-relieving surgery. Next-of-kin involvement was a major area of disagreement. A three-way split of warranted, discretionary and unwarranted transfers is suggested, as seen in Table 7, based on areas of agreement and disagreement in this Delphi survey.

### Supplementary Information

**Supplementary Material 1.**

**Supplementary Material 2.**

## Data Availability

Data used in the statistical analysis is available from the corresponding author upon reasonable request and with permission from the Norwegian Agency for Shared Services in Education and Research.

## Abbreviations

ECF: Emergency Care Facility
GP: General Practitioner
IQR: Inter-Quartile Range
LTC: Long Term Care
MAU: Municipal in-patient acute care unit
MD: Medical Doctor
NH: Nursing Home
OOH: Out-of-hours emergency primary health care centers
RN: Registered Nurse
SDM: Shared Decision Making
