# Correction: Areas of consensus on unwarranted and warranted transfers between nursing homes and emergency care facilities in Norway: a Delphi study

**DOI:** 10.1186/s12913-024-11134-5

**Published:** 2024-05-27

**Authors:** Arne Bastian Wiik, Malcolm Bray Doupe, Marit Stordal Bakken, Bård Reiakvam Kittang, Frode Fadnes Jacobsen, Oddvar Førland

**Affiliations:** 1 Centre for Care Research, West. Western, Norway University of Applied Sciences, Bergen, Norway; 2 Max Rady College of Medicine, University of Manitoba, Winnipeg, Canada; 3 National Centre for Emergency Primary Health Care, NORCE Norwegian Research Centre, Bergen, Norway; 4 University of Bergen, Bergen, Norway; 5 Department of Medicine, Haraldsplass Deaconess Hospital, Bergen, Norway

**Correction to: BMC Health Services Research (2024) 24:374**

10.1186/s12913-024-10879-3

In this article, Table 5 was published as a duplicate of Table 4 due to a typesetting mistake. The correct version of Table 5 can be found below and the article has been updated.

The publisher apologises to the authors and readers for the inconvenience caused by this error.

**Table 5:** Delphi survey on transfers between nursing homes and emergency care facilities in Norway

Delphi factor	First Round			Second Round				Third Round				
	% of score ≥ 7	IQR	Consensus Level	% of score ≥ 7	IQR	Consensus Level	Stability	% of score ≥ 7	IQR	Consensus Level	Stability	P-value < 0.05
*Transfer**** SHOULD ****happen when*:												
Condition unclear after MD assessment	43%	4	Low	40%	2	Low	No	45%	3	Low	Yes	MD (1,2,3) (-)
Condition was good before acute function decline	57%	4	Low	81%	1	**High**	**No**	**83%**	**1**	**High**	**Yes**	MD (1)(-)
Next-of-kin expresses transfer wanted	22%	3	Low	4%	1	Low	No	12%	3	Low	No	
Resident expresses transfer wanted	47%	3	Low	45%	2	Low	No	34%	2	Low	Yes	Gen (2,3) (-)
												Urb (1)(-)
A surgical operation could be pain-reliving	**90%**	**1**	**High**	**97%**	**0**	**Very high**	**Yes**	**100%**	**0**	**Very high**	**Yes**	

Table columns: round, consensus (% of score ≥ 7), inter-quartile range (IQR), stability between the round and the previous one, and significant differences (P-value < 0.05) in answers by panel characteristics gender (Gen), profession (MD) or rurality (Urb)

Bold font consensus

Minus (-) indicates lower agreement, (+) expresses higher agreement with the statement

Gender (Gen) has male set as 1

Urbanity (Urb) is a Statistics Norway measure of rurality

The questions are ranked in the order they were presented in the project proposal pre-randomized Delphi-survey

Logistic regression was used to determine significant differences in coefficients

