# Supplementary material for: Areas of consensus on unwarranted and warranted transfers between nursing homes and emergency care facilities in Norway: a Delphi study
Source: BMC Health Serv Res. 2024 Mar 26;24:374. doi: 10.1186/s12913-024-10879-3 (PMC10964583; doi:10.1186/s12913-024-10879-3)
Supplement: Supplementary file 1 — Supplementary Material 1. [file 12913_2024_10879_MOESM1_ESM.pdf]

Round 3: Transfers between Nursing Homes and Emergency Care Facilities

1. Summary of the arguments in the second round about when transfers **should not take place: Hyperlink**

At the end of each statement below, the mean value from round 2 is shown in brackets. Then, a selection of the participants' arguments in round 2 are presented.

Summary of the expert panel's answers in question 1 with distribution: hyperlink

We now ask you to answer the statements considering the answers from your fellow experts for the last time.

1. TAKE AS A STARTING POINT THE SITUATION IN THE NORWEGIAN HEALTH CARE SYSTEM IN 2023 AS YOU KNOW IT. HOW MUCH DO YOU AGREE WITH THE FOLLOWING STATEMENTS: TRANSFER OF A PATIENT WITH A LONG TERM CARE STAY IN A NURSING HOME TO AN EMERGENCY CARE FACILITY SHOULD MAINLY NOT HAPPEN:

a) When a transfer reduces residents expected lifetime significantly (Mean 8,8 in round 2)

(1) ❍ (2) ❍ (3) ❍ (4) ❍ (5) ❍ (6) ❍ (7) ❍ (8) ❍ (9) ❍

1 Completly disagree 2 3 4 5 6 7 8 Completly agree 9

Arguments on statement a) above:

"Transfers are usually very stressful for nursing home patients, they are often multimorbid and frail. Transfers should only take place when the benefit outweighs the burden."

A theoretical question that has little to do with everyday practical clinical considerations. Of course, a transfer is out of the question if it significantly reduces life expectancy. However, it is unlikely that a transfer is so burdensome. In severely frail patients, the expected health benefits from a transfer are probably small."

1 cont . ... SHOULD MAINLY NOT HAPPEN:

b) When a transfer reduces life quality for the resident in the long run

(Mean 8,6)

(1) ❍ (2) ❍ (3) ❍ (4) ❍ (5) ❍ (6) ❍ (7) ❍ (8) ❍ (9) ❍
1 Completly disagree2 3 4 5 6 7 8 Completly agree 9

Arguments on statement b) above:

"Important to think about the patient's best interests, not subject the person to unnecessary transport and examinations. Focus should be on quality of life and pain relief."

“Difficult to measure quality of life. There are many different degrees of lost quality of life that come into play when answering."

"The patient's quality of life must be the primary focus. If transfer is in conflict with this, residents should be treated where they are."

1 cont . ... SHOULD MAINLY NOT HAPPEN:

c) When a non-transfer preference was expressed in preliminary talk at the NH (Mean 8,5)

(1) ❍ (2) ❍ (3) ❍ (4) ❍ (5) ❍ (6) ❍ (7) ❍ (8) ❍ (9) ❍
1 Completly disagree2 3 4 5 6 7 8 Completly agree 9

Arguments on statement c) above:

"There may exist situations where persistently reduced quality of life and significantly reduced lifespan can trump this consideration."

1 cont . ... SHOULD MAINLY NOT HAPPEN:

d) When a transfer has significant delirium risk attached to it (Mean 7,6)

(1) ❍ (2) ❍ (3) ❍ (4) ❍ (5) ❍ (6) ❍ (7) ❍ (8) ❍ (9) ❍
1 Completly disagree 2 3 4 5 6 7 8 Completly agree 9

Arguments on statement d) above:

" “<u>has significant delirium risk attached to it</u>” is a very theoretical description. In practice, it is difficult to assess this risk, and occasionally other considerations will have to override this."

"Delirium is a real risk in almost all long-term nursing home residents."

"Risiko for forvirring i seg selv bør ikke være den viktigste faktoren for overføring om en innleggelse kan hjelpe eller forbedre den totale situasjonen til pasienten."

"Delirium is very stressful for residents and staff, and it results in a greatly reduced quality of life with long-term delirium after transfers."

<u>1 cont . ... SHOULD MAINLY NOT HAPPEN:</u>

<u>e) When the resident expresses transfer is not wanted (Mean 8,3)</u>

(1) ❍ (2) ❍ (3) ❍ (4) ❍ (5) ❍ (6) ❍ (7) ❍ (8) ❍ (9) ❍

1 Completly disagree 2 3 4 5 6 7 8 Completly agree 9

Arguments on statement e) above:

"Personen kan ha endret oppfatning siden forhåndssamtale. I tillegg kan den konkrete situasjonen ikke ha blitt vurdert den gang."

"Om pasient med samtykkekompetanse som har hatt forhåndssamtale har uttrykt at overføringer ikke ønskes tidligere, så skal det veie svært tungt og det skal gode argumenter til for ikke å følge deres ønske."

"Pasienten sitt ønske skal stå i fokus. Dersom en overføring strider med dette bør pasienten behandles der vedkommende er." The person may have changed their opinion since the preliminary interview. In addition, the specific situation may not have been assessed at the time."

"If a resident previously able to consent who has had a preliminary introductory talk when entering NH have expressed that they do not want transfers earlier, then it must weigh heavy in the consideration and good arguments must be given for not following their wish."

"The residents' wishes must be in focus. If a transfer conflicts with the resident's wishes not to be, the resident should be treated where they are."

<u>1 cont . ... SHOULD MAINLY NOT HAPPEN:</u>

<u>f) When next of kin expresses transfer is not wanted (Mean 7,1)</u>

(1) ❍ (2) ❍ (3) ❍ (4) ❍ (5) ❍ (6) ❍ (7) ❍ (8) ❍ (9) ❍
1 Completly disagree2 3 4 5 6 7 8 Completly agree 9

Arguments on statement f) above:

"Regarding the wishes of next-of-kin, they must of course be taken into account, but if they go against the wishes of the resident and staff, the latter should weigh more."

"It is the resident's wishes that must be decisive and not next-of-kin. Next-of-kin must be heard, but the resident's wishes must come first."

"The resident also usually has other wishes than next-of-kin."
"Next-of-kin often know their loved ones well and know better what the resident would choose if they could consent, like before cognitive impairment occurred due to their diagnosis."

<u>1 cont . ... SHOULD MAINLY NOT HAPPEN:</u>

<u>g) When the resident is in a palliative state (Mean 8,7)</u>

(1) ❍ (2) ❍ (3) ❍ (4) ❍ (5) ❍ (6) ❍ (7) ❍ (8) ❍ (9) ❍
1 Completly disagree2 3 4 5 6 7 8 Completly agree 9

Arguments on statement g) above:

"This depends on whether the nursing home can provide adequate palliation."

"There may be special reasons, where it is better for the patients to be transferred to hospital during the palliative phase."

"There are clear exceptions if one does not reach the goal of pain relief and other palliative measures in the nursing home."

1. Do you agree or disagree with the assessments from your fellow experts in the arguments above? (This will not be shared further with the panel as this is the final round but will be analyzed as part of the research).

____
____
____
____
____
____

Senter for omsorgsforskning • vest **SPM. 2**

1. Summary of the arguments in the second round about when transfers **should <u>take place: Hyperlink</u>**

At the end of each statement below, the mean value from round 2 is shown in brackets. Then, a selection of the participants' arguments in round 2 are presented.

Summary of the expert panel's answers in question 2 with distribution: hyperlink

We now ask you to answer the statements considering the answers from your fellow experts for the last time.

2. TAKE AS A STARTING POINT THE SITUATION IN THE NORWEGIAN HEALTH CARE SYSTEM IN 2023 AS YOU KNOW IT. HOW MUCH DO YOU AGREE WITH THE FOLLOWING STATEMENTS: TRANSFER OF A PATIENT WITH A LONG TERM CARE STAY IN A NURSING HOME TO AN EMERGENCY CARE FACILITY SHOULD MAINLY HAPPEN:

a) When the condition remain unclear after MD assessment at the Nursing Home (Mean 6,2 in round 2)

(1) ❍ (2) ❍ (3) ❍ (4) ❍ (5) ❍ (6) ❍ (7) ❍ (8) ❍ (9) ❍
1 Completly disagree 2 3 4 5 6 7 8 Completly agree 9

Arguments on statement a) above:

"If the patient is frail to begin with, it is not certain that the situation needs further clarification, as responses to will be limited in any case."

"In the terminal phase and last part of life, it is not so important to clarify diagnostics"

"Sometimes staff have to live with not being able to clarify underlying conditions."

2 cont . ... SHOULD MAINLY HAPPEN:

b) When the residents condition was good before the incidence with acute functional decline occured (Mean 7,2)

(1) ❍ (2) ❍ (3) ❍ (4) ❍ (5) ❍ (6) ❍ (7) ❍ (8) ❍ (9) ❍
1 Completly disagree 2 3 4 5 6 7 8 Completly agree 9

Arguments on statement b) above:

"Funksjonsnivå og livskvalitet i forkant av akutt sykdom sier noe om nytte og prognose, og bør vektlegges."

2 cont . ... SHOULD MAINLY HAPPEN:

c) When next of kin expresses that a transfer is wanted (Mean 4,5)

(1) ❍ (2) ❍ (3) ❍ (4) ❍ (5) ❍ (6) ❍ (7) ❍ (8) ❍ (9) ❍
1 Completly disagree 2 3 4 5 6 7 8 Completly agree 9

Arguments on statement c) above:

"Next-of-kin should not decide whether a resident should be transferred, it is the MD who has the medical responsibility who should decide that."

"Next-of-kin accept more easily that nothing can be done if they know the cause of the deterioration or why further treatment is incorrect. MDs must be supreme when it comes to deciding transfers."

"The wishes of the next-of-kin are given little weight if they are not in line with the wishes of the resident and the assessments of the MD."

"Next-of-kin should be informed so they feel seen and heard. There are cases when the resident themself do not want next-of-kin to be involved and that must be respected."

"Take the next of kin's wishes into account, but not alone, especially if the medical assessment indicates little benefit from hospitalization."

2 cont . ... SHOULD MAINLY HAPPEN:

d) When the resident expresses that a transfer is wanted (Mean 6,2)

(1) ❍ (2) ❍ (3) ❍ (4) ❍ (5) ❍ (6) ❍ (7) ❍ (8) ❍ (9) ❍

1 Completly disagree2 3 4 5 6 7 8 Completly agree 9

Arguments on statement d) above:

"Transfers must be predominantly professionally assessed and justified. The wishes of the resident should be given the most weight accordingly."

"It cannot be the case that those next-of-kin who are most intrusive receive examinations and/or unnecessary treatment for the sake of the 'tranquility of the home'."

"It is of great use if we can convince the residents they will receive just as good treatment here at the nursing home when the diagnosis is known. Then

the vast majority of them will stay, because of the more transparent and familiar surroundings, and proximity to any next-of-kin."

<u>2 cont . ... SHOULD MAINLY HAPPEN:</u>

<u>e) When a transfer for a surgical operation could be pain-reliving for the resident (Mean 8,8)</u>

(1) ❍ (2) ❍ (3) ❍ (4) ❍ (5) ❍ (6) ❍ (7) ❍ (8) ❍ (9) ❍
1 Completly disagree2 3 4 5 6 7 8 Completly agree 9

Arguments on statement e) above:

"When a surgical operation in hospital after a fracture can be pain-relieving for the resident, it is a good example of the benefit of a transfer."

"Untreated pain can lead to passivity, being bed-ridden, delirium, anxiety and other conditions"

"Transfer to hospital should take place when the hospital can provide treatment not available in a nursing home»

2. Do you agree or disagree with the assessments from your fellow experts in the arguments above? (This will not be shared further with the panel as this is the final round, but will be analyzed as part of the research.)

____
____
____
____
____
____

Do you have additional comments?

____
____

____

____

____

____

Thanks for participating!

You will receive the results on agreement and degree of agreement in all rounds of the research in the finished article when it has been published.

You will receive it by e-mail.

If you want to read a summary on the arguments in the second round, you can click here: hyperlink

If you want to read a summary on the arguments in the first round, you can click here: hyperlink

Thank you for your participating in all rounds!
