# Supplementary material for: Areas of consensus on unwarranted and warranted transfers between nursing homes and emergency care facilities in Norway: a Delphi study
Source: BMC Health Serv Res. 2024 Mar 26;24:374. doi: 10.1186/s12913-024-10879-3 (PMC10964583; doi:10.1186/s12913-024-10879-3)
Supplement: Supplementary file 2 — Supplementary Material 2. [file 12913_2024_10879_MOESM2_ESM.pdf]

Runde 3: Pasientoverføringer mellom langtidsopphold i sykehjem og akutte helsetjenester

1.Her er et sammendrag over hvilke argumenter og innspill som kom i andre runde om **når overføringer <u>ikke bør skje:</u>**

På slutten av hvert spørsmål under vises gjennomsnittet fra alle svarene i runde 2 i parentes. Deretter presenteres noen av argumentene som kom inn på påstandene i runde 2.

Sammendrag av ekspertpanelets svar i spørsmål 1 med fordeling

Vi ber deg nå svare på spørsmålene i lys av svarene fra de andre ekspertene for siste gang.

<u>1.</u> Ta utgangspunkt i situasjonen i norsk helsevesen i 2023 slik du kjenner den. Ta stilling til hvor enig du er i følgende påstander: Overføring av pasient med langtidsopphold i sykehjem til legevakt, KAD/ØHD eller sykehus <u>bør i hovedsak ikke skje:</u>

<u>a) Når overføringen antas å være så belastende at den reduserer pasientens forventede levetid vesentlig (Gj. snitt 8,8 i runde 2)</u>

(1) ❍	(2) ❍	(3) ❍	(4) ❍	(5) ❍	(6) ❍	(7) ❍	(8) ❍	(9) ❍
1 Helt uenig	2	3	4	5	6	7	8	9 Helt enig

Argumenter som kom inn på påstand a) ovenfor:

"En innleggelse er som oftest svært belastende for sykehjemspasienter, de er gjerne multimorbide og skrøpelige. Innleggelse bør bare skje når nytten overgår belastningene."

"Et teoretisk spørsmål som samsvarer lite med den kliniske hverdagen. Selvfølgelig er en overføring uaktuelt hvis det reduserer forventet levetid vesentlig. Men, det er lite trolig at en overføring er så belastende. Hos alvorlig skrøpelige er trolig forventet helsegevinst ved overføring liten."

<u>1 forts. ...bør i hovedsak ikke skje:</u>

b) Når overføringen antas så belastende at den reduserer pasientens livskvalitet på 'lang' sikt (Gj. snitt 8,6)

(1) ❍	(2) ❍	(3) ❍	(4) ❍	(5) ❍	(6) ❍	(7) ❍	(8) ❍	(9) ❍
1 Helt uenig	2	3	4	5	6	7	8	9 Helt enig

Argumenter som kom inn på påstand b) ovenfor:

"Viktig å tenke på pasientens beste, ikke utsette personen for unødvendig transport og undersøkelser. Fokus på livskvalitet og lindring."

"Vanskelig å måle livskvalitet, og her er det mange ulike grader av tapt livskvalitet som vil spille inn når man svarer."

"Pasienten sin livskvalitet skal stå i fokus. Dersom en overføring strider med dette bør pasienten behandles der vedkommende er."

1 forts. ...bør i hovedsak ikke skje:

c) Når det under forhåndssamtale med pasient/pårørende er enighet om at overføringer helst bør unngås (Gj.snitt 8,5)

(1) ❍	(2) ❍	(3) ❍	(4) ❍	(5) ❍	(6) ❍	(7) ❍	(8) ❍	(9) ❍
1 Helt uenig	2	3	4	5	6	7	8	9 Helt enig

Argumenter som kom inn på påstand c) ovenfor:

"Det kan være situasjoner hvor vedvarende redusert livskvalitet og betydelig redusert levetid kan trumfe dette hensynet."

1 forts. ...bør i hovedsak ikke skje:

d) Når overføringen medfører betydelig risiko for økt forvirring (delirium) hos pasienten (Gj.snitt 7,6)

(1) ❍	(2) ❍	(3) ❍	(4) ❍	(5) ❍	(6) ❍	(7) ❍	(8) ❍	(9) ❍
1 Helt uenig	2	3	4	5	6	7	8	9 Helt enig

Argumenter som kom inn på påstand d) ovenfor:

" "Medfører betydelig økt risiko for delirium" er en veldig teoretisk beskrivelse. I praksis er det vanskelig å bedømme denne risikoen, og av og til vil andre hensyn overstyre."

"Forvirring er en reell risiko hos nesten alle pasienter som har langtidsplass på sykehjem."

"Risiko for forvirring i seg selv bør ikke være den viktigste faktoren for overføring om en innleggelse kan hjelpe eller forbedre den totale situasjonen til pasienten."

"Delirium er svært belastende for pasient og pleiere, og det gir svært redusert livskvalitet med langvarig delirium i etterkant av innleggelse."

1 forts. ...bør i hovedsak ikke skje:

e) Når pasienten uttrykker at overføring ikke er ønskelig (Gj.snitt 8,3)

(1) ❍	(2) ❍	(3) ❍	(4) ❍	(5) ❍	(6) ❍	(7) ❍	(8) ❍	(9) ❍
1 Helt uenig	2	3	4	5	6	7	8	9 Helt enig

Argumenter som kom inn på påstand e) ovenfor:

"Personen kan ha endret oppfatning siden forhåndssamtale. I tillegg kan den konkrete situasjonen ikke ha blitt vurdert den gang."

"Om pasient med samtykkekompetanse som har hatt forhåndssamtale har uttrykt at overføringer ikke ønskes tidligere, så skal det veie svært tungt og det skal gode argumenter til for ikke å følge deres ønske."

"Pasienten sitt ønske skal stå i fokus. Dersom en overføring strider med dette bør pasienten behandles der vedkommende er."

<u>1 forts. ...bør i hovedsak ikke skje:</u>

<u>f) Når pasientens pårørende uttrykker at overføring ikke er ønskelig (Gj.snitt 7,1)</u>

(1) ❍	(2) ❍	(3) ❍	(4) ❍	(5) ❍	(6) ❍	(7) ❍	(8) ❍	(9) ❍
1 Helt uenig	2	3	4	5	6	7	8	9 Helt enig

Argumenter som kom inn på påstand f) ovenfor:

"Når det gjelder pårørendes ønsker, skal de selvfølgelig hensyntas, men hvis de går på tvers av pasientens og behandlers ønske bør disse veie tyngst."

"Det er pasientens ønsker som må være avgjørende og ikke pårørende. Pårørende skal bli hørt, men er pasientens ønsker som må komme først."

"Pasienten har også som regel andre ønsker enn pårørende."

"Pårørende kjenner ofte godt sine nærmeste og vet bedre hva de ville valgt dersom de hadde samtykkekompetanse, slik de hadde før kognitiv svikt grunnet deres diagnose."

<u>1 forts. ...bør i hovedsak ikke skje:</u>

<u>g) Når pasienten er i livets avslutning/palliativ fase (Gj.snitt 8,7)</u>

(1) ❍	(2) ❍	(3) ❍	(4) ❍	(5) ❍	(6) ❍	(7) ❍	(8) ❍	(9) ❍
1 Helt uenig	2	3	4	5	6	7	8	9 Helt enig

Argumenter som kom inn på påstand g) ovenfor:

"Dette er avhengig av om sykehjemmet kan gi tilstrekkelig palliasjon."

"Det kan være spesielle årsaker som gjør at det beste for pasientene er å overføres til sykehus i en palliativ fase."

"Det finnes klare unntak dersom man ikke kommer til målet med smertelindring og andre palliative tiltak på sykehjemmet."

1. Er du enig eller uenig med vurderingene fra ekspertpanelet i påstandene over? (Dette vil ikke bli delt med panelet siden dette er siste runde, men vil bli analysert for forskningsrapporten.)

____
____
____
____
____
____

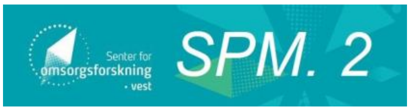

2. Her er et sammendrag over hvilke argumenter og innspill som kom i andre runde om **når overføringer bør i hovedsak skje:**

På slutten av hvert spørsmål under vises gjennomsnittet fra alle svarene i runde 2 i parentes. Deretter presenteres noen av argumentene som kom inn på påstandene i runde 2.

Sammendrag av ekspertpanelets svar i spørsmål 2 med fordeling

Vi ber deg nå svare på spørsmålene i lys av svarene fra de andre ekspertene for siste gang.

2. Ta utgangspunkt i situasjonen i norsk helsevesen i 2023 slik du kjenner den. Ta stilling til hvor enig du er i følgende påstander: Overføring av pasienter med langtidsopphold i sykehjem til legevakt, KAD/ØHD eller sykehus bør i hovedsak skje:

a) Når pasientens tilstand er uavklart selv etter legeundersøkelse på

sykehjemmet (Gj. snitt 6,2 i runde 2)

(1) ❍	(2) ❍	(3) ❍	(4) ❍	(5) ❍	(6) ❍	(7) ❍	(8) ❍	(9) ❍
1 Helt uenig	2	3	4	5	6	7	8	9 Helt enig

Argumenter som kom inn på påstand a) ovenfor:

"Dersom pasienten er skrøpelig i utgangspunktet er det ikke sikkert at situasjonen trenger avklaring, da det uansett vil være begrenset hva man kan gjøre med det man eventuelt finner."

"I terminal fase og siste del av livet er det ikke så viktig å avklare diagnostikk"

"En må av og til leve med at en ikke får avklart underliggende tilstander."

2 forts. ...bør i hovedsak skje:

b) Når pasientens generelle allmenntilstand var god før den akutte hendelsen oppsto med akutt funksjonsfall (Gj.snitt 7,2)

(1) ❍	(2) ❍	(3) ❍	(4) ❍	(5) ❍	(6) ❍	(7) ❍	(8) ❍	(9) ❍
1 Helt uenig	2	3	4	5	6	7	8	9 Helt enig

Argumenter som kom inn på påstand b) ovenfor:

"Funksjonsnivå og livskvalitet i forkant av akutt sykdom sier noe om nytte og prognose, og bør vektlegges."

2 forts. ...bør i hovedsak skje:

c) Når pasientens pårørende ønsker at pasient skal legges inn (Gj.snitt 4,5)

(1) ❍	(2) ❍	(3) ❍	(4) ❍	(5) ❍	(6) ❍	(7) ❍	(8) ❍	(9) ❍
1 Helt uenig	2	3	4	5	6	7	8	9 Helt enig

Argumenter som kom inn på påstand c) ovenfor:

"Pårørende skal ikke bestemme om pasient skal overføres, det er legen som har det medisinske ansvar som bør bestemme det."

"Pårørende aksepterer lettere at ingenting kan gjøres dersom de vet årsak til forverring eller hvorfor videre behandling ikke er riktig. Lege må være suveren når det gjelder overføringer."

"Pårørendes ønske vektes lite dersom de ikke er i tråd med pasientens ønske og den medisinsk ansvarliges vurderinger."

"Pårørende bør informeres slik at de føler seg sett og hørt. Det er tilfeller når pasient selv ikke ønsker at pårørende er involvert og det må respekteres."

"Ta hensyn til pårørendes ønsker, men ikke alene, spesielt dersom den medisinske vurderingen tilsier lite nytte av innleggelse."

<u>2 forts. ...bør i hovedsak skje:</u>

<u>d) Når pasienten selv ønsker innleggelse (Gj.snitt 6,2)</u>

(1) ❍	(2) ❍	(3) ❍	(4) ❍	(5) ❍	(6) ❍	(7) ❍	(8) ❍	(9) ❍
1 Helt uenig	2	3	4	5	6	7	8	9 Helt enig

Argumenter som kom inn på påstand d) ovenfor:

"Hovedsakelig må innleggelse faglig vurderes og begrunnes. Pasientene sine ønsker bør vektes mest deretter."

"Det kan ikke bli slik at de som er mest påtrengende får undersøkelser og eller

unødig behandling for "husfredens skyld"."

"Det er til stor hjelp om vi kan overbevise pasientene om at de får like god behandling her på sykehjemmet når diagnosen er kjent. Da vil de aller fleste bli, på grunn av mer oversiktlig og kjent avdeling, og nærhet til eventuelle pårørende."

<u>2 forts. ...bør i hovedsak skje:</u>

<u>e) Når en kirurgisk operasjon på sykehus etter brudd kan være smertelindrende for pasienten (Gj.snitt 8,8)</u>

(1) ❍	(2) ❍	(3) ❍	(4) ❍	(5) ❍	(6) ❍	(7) ❍	(8) ❍	(9) ❍
1 Helt uenig	2	3	4	5	6	7	8	9 Helt enig

Argumenter som kom inn på påstand e) ovenfor:

"Når en kirurgisk operasjon på sykehus etter brudd kan være smertelindrende for pasienten er det et godt eksempel på nytte ved overføring."

"Ubehandlet smerte kan føre til passivitet, sengeleie, delirium, engstelse og andre tilstander"

"Overføring til sykehus bør skje når sykehuset kan gi en behandling som ikke kan gis på sykehjem"

2. Er du enig eller uenig med vurderingene fra ekspertpanelet i påstandene over? (Dette vil ikke bli delt med ekspertpanelet siden dette er siste runde, men vil bli analysert for forskningsrapporten.)

____
____
____
____
____
____

Har du ytterligere kommentarer til undersøkelsen eller tanker om temaet?

____
____
____
____
____
____

Tusen takk for ditt bidrag!

Du vil få en tilbakemelding på eventuell enighet og grad av enighet i alle rundene i forskningsartikkelen når den er ferdigstilt.
Du vil få den tilsendt på e-post.

Dersom du vil lese oppsummering fra alle spørsmålene i andre runde kan du klikke her:

Rapport fra ekspertpanel andre runde

Dersom du vil lese oppsummering fra alle spørsmålene i første runde kan du klikke her:
Rapport fra ekspertpanel første runde

Tusen takk for din deltakelse i samtlige runder!
